# Experimental Study on Restoration Materials of Newly Earthen Ruins under Different Slaking Times

**DOI:** 10.3390/ma15124356

**Published:** 2022-06-20

**Authors:** Jianwei Yue, Wenhao Li, Xiang Zhu, Qingmei Kong, Xuanjia Huang, Xue Yang, Zhiguang Han

**Affiliations:** 1 School of Civil Engineering and Architecture, Henan University, Kaifeng 475004, China; yjw@vip.henu.edu.cn (J.Y.); l308727900@126.com (W.L.); hxj_henu@126.com (X.H.); yx1391989351@126.com (X.Y.); hanzhiguang01@163.com (Z.H.); 2 Key Laboratory for Restoration and Safety Evaluation of Immovable Cultural Relics in Kaifeng City, Kaifeng 475004, China; 3 Key Research Institute of Yellow River Civilization and Sustainable Development, Henan University, Kaifeng 475004, China

**Keywords:** earthen ruins, slaking time, restoration materials, compressive strength, pH value

## Abstract

The newly repaired Kaifeng City Wall has serious cracks, shedding and other issues on the surface, which constitute a significant problem. It is of great significance for the restoration of Kaifeng City Wall to explore the repair materials and techniques suitable for Kaifeng City Wall. The pH, particle gradation, compressive strength and SEM were tested on soil samples with different lime and MgO contents under different slaking times. With the increase of slaking time, the pH value first increased and then decreased. The relationship between pH value and strength showed three stages. The strength of lime-containing soil samples increased first, then decreased and then increased. The MgO content of soil samples showed an opposite trend. The particle gradation was significantly improved with increasing aging time. The main reason for the reduction of soil strength is the calcium carbonate crystals and magnesite microcracks produced by lime and MgO in the later stage of slaking.

## 1. Introduction

**Figure 1:**
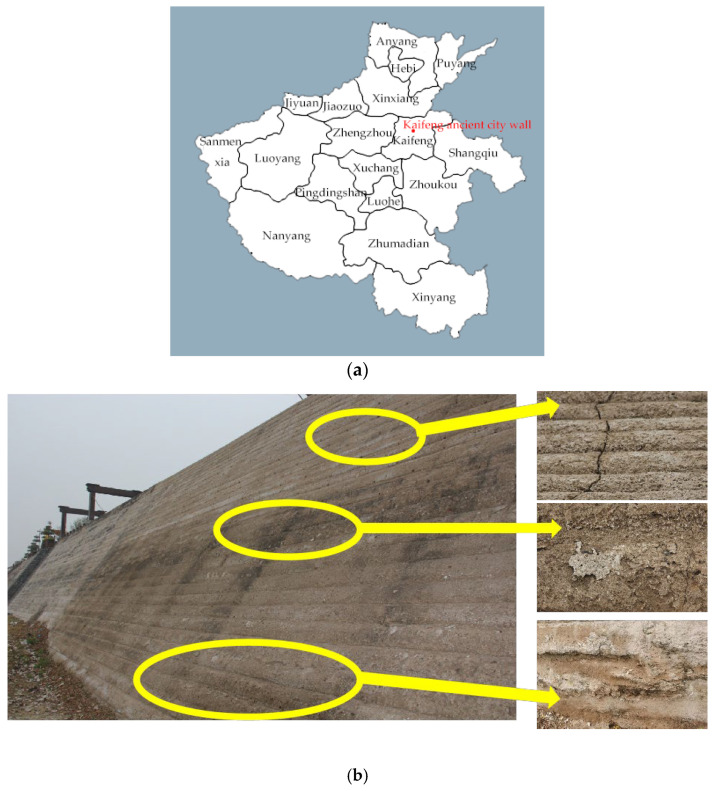
Current state of newly earthen ruins. (**a**) Kaifeng Ancient City Wall location; (**b**) current state of Kaifeng Ancient City Wall.

Kaifeng City Wall was built in the second year of Emperor Dezong’s construction of the Tang Dynasty (AD 781). It is located in Kaifeng City, Henan Province, with a total length of 14.4 km. It is the second-largest existing Ancient City Wall building in China after Nanjing City Wall. It is a key cultural relic protection unit in China. Due to the flooding of the Yellow River, five layers of ancient city walls were stacked under the city walls. The object of research in this paper is the artificial rammed-earth city walls built during the Qing Dynasty in China. With the continuous development of urbanization and people’s increasing awareness of historical structure protection, the ancient ruins in the city are being paid more and more attention by people, and the repair work of earthen ruins has been extensive. Although the Kaifeng City Wall has been repaired many times, the effect is still not ideal. Located in the Central Plains, Kaifeng belongs to a typical temperate continental climate region, with high temperature and rain in summer, low temperature and little rain in winter, long light exposure time and large temperature difference in spring and autumn festivals. In such a climate environment, the ruins themselves are prone to expansion and cracking, mechanical properties decline, and overall structural damage [1]. However, there are still construction techniques and materials used during the repair so that the Kaifeng City Wall will appear with “warped skin” and “cracks” that “fall off”, among other phenomena on the surface, soon after the repair is completed (Figure 1). 

Most ancient city walls in China used lime as a binder [2]. In ancient times, people had poor construction technology and long construction periods, which was in line with the slow reaction rate of lime carbonization in the air, making them have the characteristics of high strength and good durability after completion. However, with the continuous development of technology, the construction speed on-site is fast, and the construction period is short. According to the previous slaking time, it is difficult to meet the requirements of construction speed, and the quality after completion cannot reach the expected result. The surface of the treated site is easy to whiten and falls off in layers. Therefore, it is urgent to explore the impact of slaking time on material properties or explore a material that satisfies the repair requirements and current construction speed to repair the earthen ruins.

The results of many researchers have promoted research on the carbonization mechanism of lime and MgO and the degree of carbonization on their mechanical properties to a certain extent. C. Unluer conducted exploratory, experimental research on simulated sand and silt solidified by MgO carbonization and found that MgO solidified soil has the advantages of a short construction period and high strength compared with cement solidified soil and verified the feasibility of active MgO in the field of soil sample reinforcement [3]. Padmaraj et al. studied the carbonization mechanism of lime-stabilized silty clay from the chemical and microscopic perspectives with a lime content of 4% and 8%, and proposed that the carbonization time is too long, which will damage its strength, but this study did not include higher lime dosage samples [4]. E. Vitale et al. also proposed that carbonation affects the chemical and mineralogy evolution of lime-treated soil, depending on the time scale of the reaction mechanism, indicating that different slaking times have a great impact on the performance of lime-treated soil [5]. 

Hyun et al. observed the intercalation of water between Ca (OH)_2_ layers, clarified the importance of Ca(OH)_2_ in carbonation reaction, and proposed the interaction between water and Ca(OH)_2_. The role is crucial for controlling the kinetics and thermodynamics of the Ca(OH)_2_ carbonization reaction [6]. Cai et al. studied the effects of MgO content and activity on engineering properties by conducting no lateral compression test on the samples under a constant carbonization pressure of 200 kPa, and pointed out that the MgO content and carbonization time had an impact on the engineering properties of soil [7]. The critical content of MgO was about 25%, and the critical carbonization time was about 10 h, but they accelerated the carbonization of the sample and did not mention the performance of MgO carbonization in the natural state. Unlue studied the effect of particle size on the carbonization of MgO blocks through the accelerated carbonization of four samples with different aggregate profiles at 20% CO_2_ concentration for 28 days and proved that the degree of CO_2_ diffusivity mainly depends on the pore connectivity rather than the total pore volume, but does not take into account the effect of carbonation at lower concentrations of CO_2_ [8]. Liu et al. improved the compressive strength of electrolytic manganese waste residues by solidifying electrolytic manganese residues with four kinds of magnesium oxide content (5%, 10%, 15%, 20%) and proved that with the participation of microorganisms, the active magnesium oxide mixed with the dosage has a significant effect on the solidification effect of electrolytic manganese waste residue. The higher the dosage, the better the effect [9]. 

Cao et al. conducted a series of physical and mechanical tests on plain soil and improved soil with different lime content (0% to 8%) and found that there is an optimal lime content ratio. In the actual construction process, the soil should be comprehensively considered. According to the properties and design indicators of the body itself, the appropriate amount of lime is selected, but the changes at the microscopic level are not considered [10]. Wang et al. selected two large industrial waste residues, slag and fly ash, as solidifying materials, respectively mixed with soil samples, and after sample preparation, CO_2_ was introduced to conduct carbonization experiments, and the effect and mechanism of CO_2_ carbonized slag CaO-MgO to strengthen soil were explored. It is found that CO_2_ carbonization has a significant improvement effect on soil consolidation, and CO_2_ carbonization promotes the formation of carbonate crystals (CaCO_3_, MgCO_3_) [11]. 

Zhang et al. used steel powder as the main cementitious material, mixed with MgO, CaO and cement to prepare mortar specimens, and tested their compressive strength and microstructure after carbonization curing. It is found that active MgO and CaO are beneficial to improving the strength of cement mortar carbonized samples, and the strength reaches the highest when the mass ratio of MgO/CaO is 3:1, which also shows that MgO and CaO have similar characteristics [12]. In the case of good quicklime activity, Gu Li et al. explored the three operating conditions of initial water temperature, water to lime mass ratio, and slaking time to find the optimal conditions. However, the longest slaking time was only 12 h, and the longer slaking time was not considered [13]. 

Wei et al. used SEM and other technical means to discuss the process mechanism of lime slaking (0 d~76 d) and the feasibility of its application in cultural relics protection. They found that slaked lime has good rheology, water retention, dense adhesion and the microscopic mechanism of high reactivity has laid a certain foundation for the protection of cultural relics, but the role of lime in the early stage of slaking has not been considered [14].

In summary, most scholars pay attention to the properties of MgO while studying CaO, which shows that the two materials are similar, and the experimental research on the carbonation mechanism and micro model of MgO and CaO is relatively sufficient. However, most scholars use accelerated carbonization in their experiments, and there are few studies on the effect of early slaking on the properties of the two materials in the natural state of low concentration CO_2_.

Therefore, this study takes the restoration of the Kaifeng City Wall as the object, and mainly silty clay, adding MgO and lime, two alkaline oxides, respectively, as soil site restoration materials, and studies the influence on its performance by designing different slaking times. The research results provide a certain experimental basis for the restoration and safety evaluation of the Kaifeng Ancient City Wall.

## 2. Materials and Methods

### 2.1. Experiment Material

#### 2.1.1. Silty Clay

**Figure 2:**
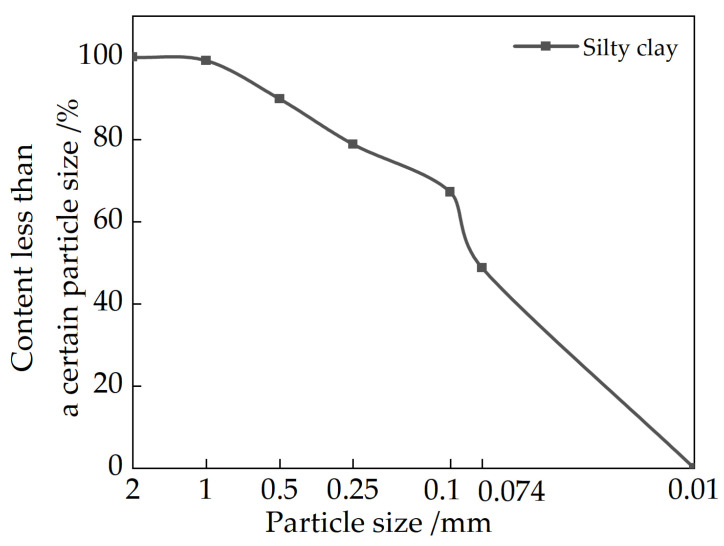
Particle gradation of silty clay.

**Figure 3:**
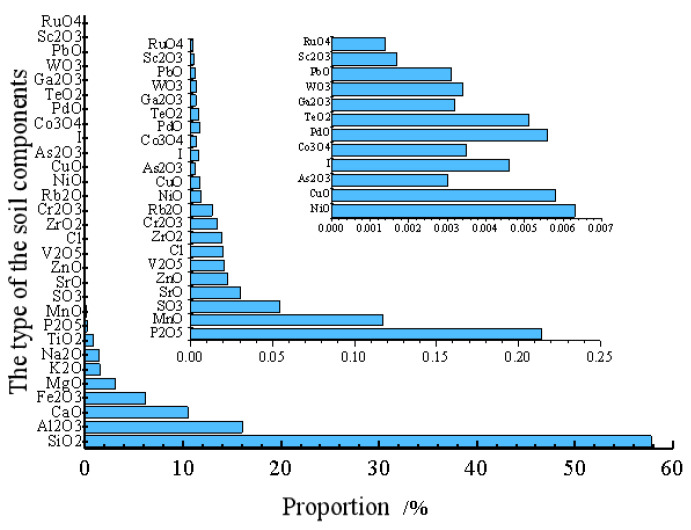
Main chemical composition of silty clay.

The soil used for the test is near the Kaifeng Ancient City Wall, and the geotechnical test is carried out according to the Geotechnical Engineering Test Method and Criterion (GB/t50123-2019). Its chemical composition content has been analyzed by specific chemical composition; the results are shown in Table 1 and Figure 2 and Figure 3.

#### 2.1.2. Quicklime

The lime used in the test is Huihui lime, which was purchased from Shanggao Tengshun Company in Xinyu, China. The main physical and chemical indexes are as follows: the relative molecular weight is 56.08, the CaO content is not less than 98%, and the loss on ignition is 2%. Mixing lime with soil will produce a chemical reaction with water in the soil and CO_2_ in the air:

(1) $$\mathrm{CaO}+H_{2}O\to \mathrm{Ca}{\left(\mathrm{OH}\right)}_{2}$$

(2) $$\mathrm{Ca}{\left(\mathrm{OH}\right)}_{2}+{\mathrm{CO}}_{2}\to {\mathrm{CaCO}}_{3}+H_{2}O\;$$

Both of these reaction products have an effect on the strength of the soil sample.

#### 2.1.3. MgO

MgO is produced by Tianjin Zhiyuan Chemical Reagent Co in Tianjing, China. The main physicochemical indexes are as follows: relative molecular weight is 40.30, effective content of MgO ≥ 98%, weight loss by burning is 2%, easy to absorb CO_2_ and water in the air gradually becomes one or several kinds of alkali magnesium carbonate. The main chemical reactions took place as follows:

(3) $$\mathrm{MgO}+H_{2}O\to \mathrm{Mg}{\left(\mathrm{OH}\right)}_{2}$$

(4) $$\mathrm{Mg}{\left(\mathrm{OH}\right)}_{2}+{\mathrm{CO}}_{2}+2H_{2}O\to {\mathrm{MgCO}}_{3}\cdot H_{2}O\;$$

(5) $$5M{\left(\mathrm{OH}\right)}_{2}+4{\mathrm{CO}}_{2}+H_{2}O\to \left(\mathrm{Mg}\right)5\left({\mathrm{CO}}_{3}\right)4{\left(\mathrm{OH}\right)}_{2}\cdot 5H_{2}O$$

(6) $$5\mathrm{Mg}{\left(\mathrm{OH}\right)}_{2}+4{\mathrm{CO}}_{2}\to \left(\mathrm{Mg}\right)5\left({\mathrm{CO}}_{3}\right)4{\left(\mathrm{OH}\right)}_{2}\cdot 4H_{2}O\;$$

### 2.2. Specimen Preparation

#### 2.2.1. Design of the Experiment

**Figure 4:**
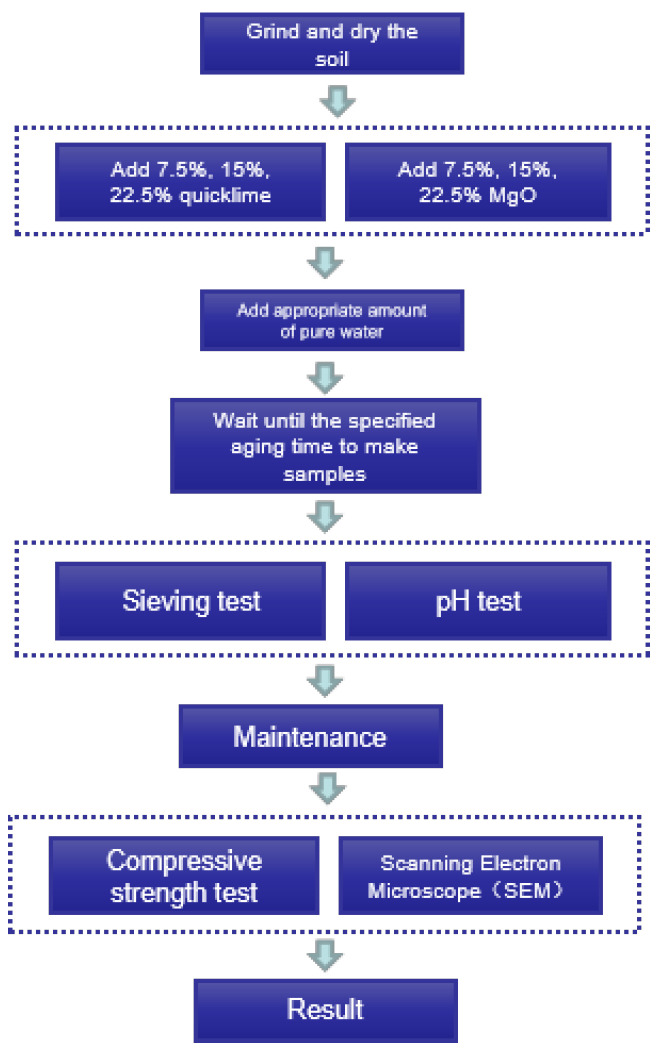
Production process.

In order to make this test widely applicable and in accordance with the effect of MgO admixture on the carbonation effect [15] combined with the dosages more commonly used, the admixture of both lime and MgO were selected as 7.5%, 15% and 22.5%, respectively. In accordance with the standard of each soil sample, each production was compacted according to the specification requirements, and the water content was the optimal moisture content. However, MgO and calcium oxide will have a digestion reaction when contacting with water, which will consume the water in the soil sample and reduce the water content of the soil. According to the calculation, it is known that the theoretical water consumption for CaO hydration is 32.16% of the mass of CaO and the theoretical water consumption for MgO hydration is 45% of the mass of MgO [16]. The digestion reaction was an exothermic reaction; the exotherm of CaO is more obvious. In order to ensure the different content of MgO, the soil samples of lime had the same water content. The determination of the test water consumption was the water required for optimal moisture content + water used for digestion. The specific process is shown in Figure 4.

#### 2.2.2. Soil Samples Preparation

Before the test, the obtained site soil was dried and crushed, passed through a 2 mm sieve and dried, and mixed with different amounts of MgO, lime and silty clay, respectively. The weighed water was poured into the mixture and mixed again, and left to stand. We then waited until different slaking times were achieved, and the pH value of the soil mix was taken via the sieve test. The compaction method for sample making was used, with three parallel samples taken for each slaking time. After the soil samples were taken, they were maintained for seven days; the first three days at constant humidity and the last four days at normal temperature. After the soil samples were maintained, they were tested for compressive strength. In order to better explore the characteristics of the repair material, we also carried out the same preparation method for the silty clay used in the experiment and tested it as a control group. The experimental design is shown in Table 2.

### 2.3. Test Method

To better investigate the effect of slaking on two materials, lime and MgO, we used a pH test, sieving test, and compressive test.

#### 2.3.1. pH Test

When the set slaking time was reached, 100 g of mixed soil was taken and a Shanghai Sanxin pen-type pH meter pH5S was used to measure the pH value (potential of hydrogen) of the mixed soil restoration material (accuracy is ±0.01), and each measurement was inserted into the test. After the reading was stabilized, the measured pH value was measured twice. Each sample was measured three times, and the average value was recorded.

#### 2.3.2. Sieving Test

After taking 300 g of soil and drying, the soil sample was placed into the test screen (Shanghai Dongxing Building Materials Testing Equipment Co., Ltd., Shanghai, China.) and placed on the vibrating screen machine (Shock-Type Standard Vibrating Screen Machine, Shanghai Dongxing Building Materials Testing Equipment Co., Ltd.). The sample was shaken for 15 min and the screen was removed. The samples on each screen were weighed, respectively, and recorded.

#### 2.3.3. Compressive Strength Test

A layer of lubricant was applied to both ends of the cured samples and placed on a TSZ (Instrument model) automatic triaxial testing machine produced by Nanjing Ningxi Soil Instrument Co, Ltd., Nanjing, China. The resolution of the pressure sensor was 0.01 kN, and the resolution of the displacement sensor was 0.001 mm. The instrument started to pressurize, then was turned off, and after the sample was completely destroyed, the pressure was released, and the sample was taken out. The data on the instrument was exported and recorded [17].

Through these three indicators, the optimal slaking time of soil materials for restoration sites is determined. In order to better reduce the human error caused by the test, all test methods were strictly carried out in accordance with the Standard for Geotechnical Testing Method (GB/T50123-2019), and three parallel samples were set for all tests.

## 3. Results

### 3.1. Effect of Slaking Time on pH

**Figure 5:**
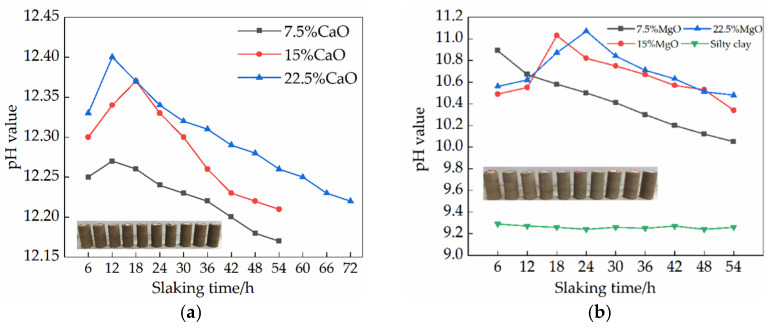
Effects of different slaking times on pH value of repair materials: (**a**) CaO repair materials; (**b**) MgO repair materials.

Two basic oxides, CaO and MgO, were added to the soil samples, respectively, and CO_2_ present in the air was an acidic oxide, so the hydrolysis and carbonization of CaO and MgO must have caused a change in the pH of the soil samples. Figure 5 shows that the dissociation reaction of CaO and MgO leads to an increase in the concentration of Ca^2+^ and Mg^2+^ in the pore water, causing an increase in pH [18]. Since CaO and MgO hydrate to produce Ca(OH)_2_ and Mg(OH)_2_, and the higher the admixture, the more products, the more alkaline the soil sample [19]. With the increasing amount of MgO and CaO doping, the time for the basic completion of the alkaline oxide hydration reaction increased accordingly.

The contents of 5%, 15%, and 22.5% of MgO and CaO reached their peaks at different slaking times, respectively. This is because the soil of the Kaifeng Ancient City Wall site is unsaturated soil, and then the added water cannot react with CaO and MgO directly—it has to react by absorbing the free water in the soil sample with the increasing amount of admixture, the percentage of alkaline oxide in the soil sample increases, the free water in the soil sample decreases and the reaction rate slows down. The amount of generated oxide increases, and the concentration becomes higher, so the pH peak point gradually increases with the increase of doping. Meanwhile, the generated Ca(OH)_2_ and Mg(OH)_2_ absorb CO_2_ and water in the air and gradually transform into alkali carbonate, and the carbonation reaction starts to dominate with time.

The reduction in the total pore volume and the gas permeability was poor in the soil sample during carbonation due to the production of alkali carbonates, which hinders the transport channels for CO_2_ and will not allow better contact of the hydration products with CO_2_, resulting in a slow carbonation process and a reduced rate of pH decrease later in the process [20]. However, since the calcium and magnesium carbonate salts produced by carbonation both have a medium-strong base and weak acid salts, the measured pH values are alkaline (pH of magnesium carbonate trihydrate in carbonated water is about 9.2) [10]. Although the pH of the soil sample decreased continuously with increasing carbonation time during carbonation, the pH of the soil sample mixed with MgO remained above 10.1 after 54 h of slaking, and the pH of the CaO soil sample remained above 12.15, both maintaining a high alkaline level [21]. However, the pH value of the soil samples with 15% MgO content decreased significantly after slaking for 54 h, which may be due to the error of the instrument itself during the measurement and the error caused by the inhomogeneous mixing of the soil.

### 3.2. Effect of Slaking Time on Repair Materials Strength

**Figure 6:**
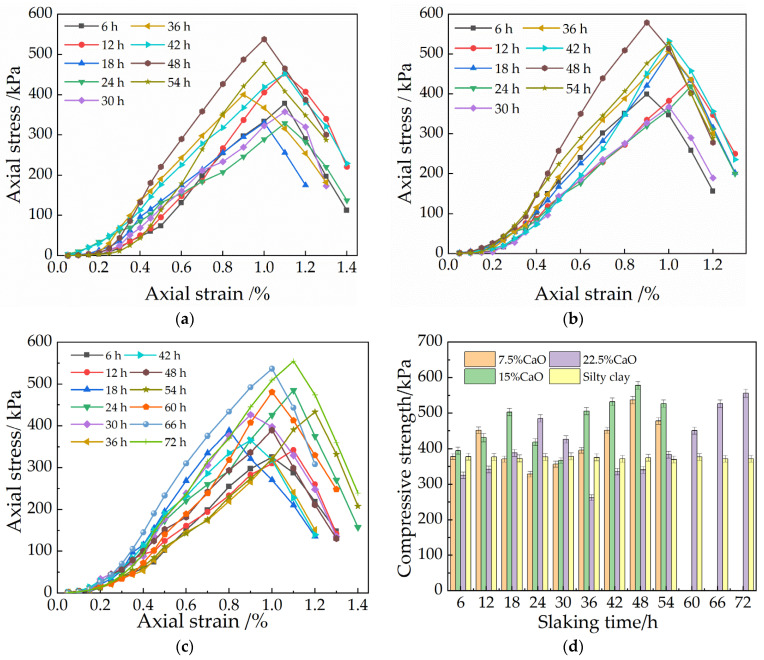
Axial stress–strain relationship curves of repair materials with different CaO contents: (**a**) 7.5%CaO; (**b**) 15%CaO; (**c**) 22.5%CaO. (**d**) Compressive strength of CaO repair material.

**Figure 7:**
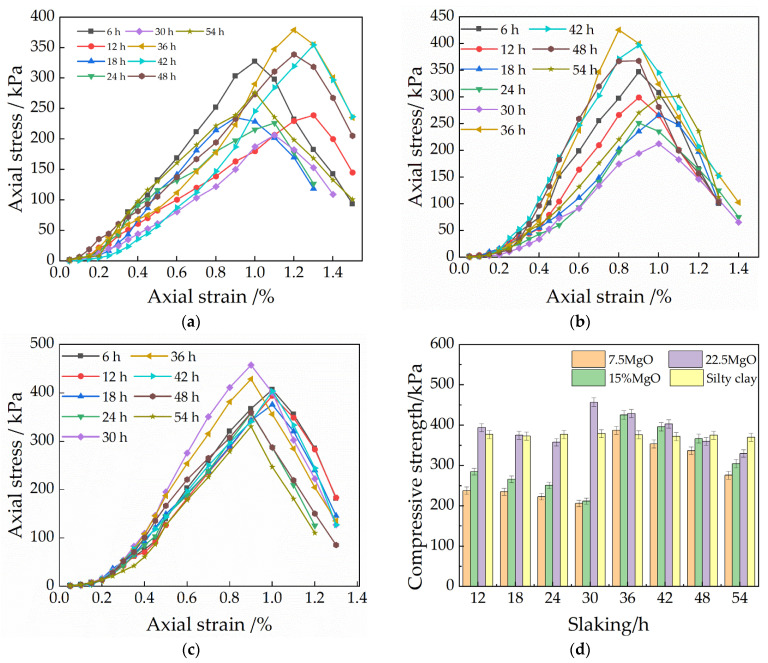
Axial stress–strain relationship curves of repair materials with different MgO contents: (**a**) 7.5% MgO; (**b**) 15% MgO; (**c**) 22.5% MgO; (**d**) Compressive strength of MgO repair material.

Figure 6 and Figure 7 show the stress–strain curves of the samples with three different dosages of CaO and MgO under different slaking times. The stress–strain process of the specimen includes two stages: Stage I is the nonlinear rising stage of the stress–strain curve, at which time the stress σ increases gradually with the strain ε; Stage II is the steep drop stage of the stress–strain curve, where the stress σ increases. The sudden decrease with the increase of strain ε is also the failure stage of the specimen. When the stress–strain curve reaches its peak value, the peak value is the ultimate strength of the sample, and the sample breaks rapidly, and the corresponding strain is the failure strain. The strain corresponding to the peak stress of the specimen is defined as the failure strain. It can be seen from the figure that the change of the slaking time has a significant influence on the unconfined compressive strength of the repaired soil site materials, but there are differences in the growth trend of the strength of the two repaired materials with the slaking time.

#### 3.2.1. Effect of Ageing Time on the Strength of CaO Repair Materials

It can be found from Figure 6 that with the increase in slaking time, the ability of the sample to resist damage increases first, then decreases and then increases. The axial stress reaches the maximum value when the slaking time of the three dosages is 48 h, 48 h and 72 h—that is, the unconfined compressive strength reaches the maximum value. It can be seen from Figure 6 that with the increase in slaking time, the failure strains of the three dosages at the final slaking time changed from 1.1%, 0.95%, and 0.97% to 1.0%, 1.0%, and 1.15%, respectively. Tang et al. [22] believed that the deformation characteristics of soil samples could be characterized by failure strain. The larger the failure strain, the stronger the soil sample toughness. This shows that with the increase in the slaking time, the higher content of lime can increase the toughness of the sample after a longer slaking time. The failure strains at the maximum stress in different slaking times are 1.0%, 0.9%, and 1.15%, respectively. It shows that the toughness of the samples with 7.5% and 15% lime content decreases when the strength is the highest, while the toughness of the samples with 22.5% lime content increases. The strength of the soil sample with three different CaO doping levels increases a little in the early stage [23]. At 6 h of slaking, the strength of the 15% CaO-doped soil sample significantly increased, reaching 430 kPa, and thereafter, the strength of the soil sample with different contents increased and reached the first peak point at 12, 18, and 24 h, respectively, the higher the content, the longer the time required. However, the strength decreases after reaching the first peak point, and the strength of the soil sample with 22.5% CaO admixture reaches the lowest after 36 h of slaking, which is only 255 kPa, 85% of the strength of the silty clay. With the increase in slaking time, the strength of the soil sample will increase again. and the strength of the mixes with 7.5%, 15%, and 22.5% CaO doping reached the peak point of the second stage at 48 h, 48 h, and 72 h, respectively, 5875 kPa, 593 kPa, and 550 kPa, which is 60% higher than that of the silty clay at the highest. This is because as the carbonation continues, a denser gel mass is formed and the pores are filled with calcium carbonate, which further increases its strength [24]. The peak points reached in the second stage are higher than the strength of the previous stage, which shows that the strength of the soil sample still increases with the increasing slaking time and also shows the necessity of slaking.

#### 3.2.2. Effect of Ageing Time on the Strength of MgO Repair Materials

It can be seen from Figure 7 that with the increase of slaking time, the ability of the sample to resist damage shows a change rule that firstly decreases, then increases, and then decreases. The axial stress reaches the maximum value; that is, the unconfined compressive strength reaches the maximum value. It can be seen from the stress–strain curve that with the increase of MgO content, the final strain of the sample becomes smaller and smaller, showing more and more brittle characteristics. The failure strains in different slaking times range from 1.0%, 1.0%, and 1.0% for 6 h of slaking to 1.0%, 1.0%, and 0.9% for 54 h of slaking. Resilience has little effect. The strength of the three different MgO-doped soil samples gradually decreased in the first period with the increase in slaking time. The strength of all soil samples ranged from 320 to 350 kPa at 6 h of slaking, indicating that the strength of soil samples was not greatly affected at the early stage of slaking. After this stage, the strength decreases, and the strength of 7.5% MgO admixture reaches the lowest after 30 h of slaking—only 200 kPa—and the strength decreases by 60%, which is the group with the highest degree of decrease among the three contents and is 70% of the strength of the silty clay. It can be seen that the strength of the soil sample with the addition of MgO is not high in the early stage and does not reach the compressive strength of the silty clay, which is far from the expected strength requirement [25]. However, when slaked from 24 h to 30 h, the strength of the soil sample increases significantly, with the content of 7.5%, 15%, and 22.5% MgO reaching peak points at 36 h, 36 h, and 30 h of slaking with strengths of 395 kPa, 433 kPa, and 468 kPa, respectively, up to 60% higher than the strength of the silty clay. This is because the generated carbonation products facilitate the filling of the soil sample pores, further compressing the pores and cementing the particles, which increases the strength [26]. However, with the continued increase of this slaking time, the strength all produced a tendency to decrease, and before 48 h, the strength could all reach a high level. However, after 54 h of slaking, the strength decreased compared to that after 6 h of slaking, and 22.5% MgO was even only at 265 kPa. It can be seen that the continuation of slaking at this time will have a negative impact on the strength. This is related to the fact that the carbonization reaction of MgO is a volume expansion process [27]. On the other hand, the water film formed on the surface of Mg(OH)_2_, which is slightly soluble in water, will also hinder the reaction of Mg(OH)_2_ with CO_2_.

### 3.3. Effect of Slaking Time on Particle Gradation

#### 3.3.1. Effect of Slaking Time on the Gradation of CaO Repair Materials Particles

**Figure 8:**
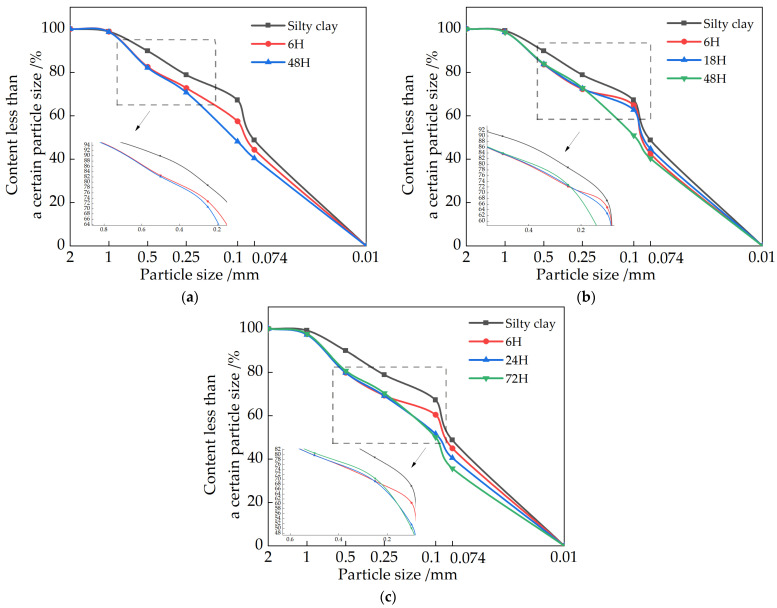
Particle gradation of repair materials with different CaO contents: (**a**) 7.5%CaO; (**b**) 15%CaO; (**c**) 22.5%CaO.

Figure 8 shows the particle gradation curves for different slaking times with different CaO contents. As shown in Figure 8, compared to the initial sample particle gradation curves, in all nine groups of different slaking time scenarios, the most significant changes in particle gradation were observed at the beginning of mixing and at the highest strength for all nine samples. The specific gravity of particles in the 0.5 mm to 1 mm particle size range increased from 9% to 16% and decreased from 18% to 8.12% in the 0.1 to 0.074 mm particle size range for 7.5% CaO at 6 h of slaking. The 15% and 22.5% contents were essentially the same as 7.5%, although the change from 0.5 mm to 1 mm was essentially the same. However, the content of 0.1 mm to 0.074 mm particle range was almost the same as that of the silty clay, indicating that the low content of CaO reacts faster in the soil during the pre-slaking period and has a greater effect on the particle gradation of the soil samples. 

However, for the 15% and 22.5% CaO soil samples, the sudden change in the 0.1 mm to 0.074 mm particles occurred at 18 h and 24 h, decreasing from 18.46% to 5.03% and 5.68%, respectively—a decrease of about 13%. During this slaking time, the soil particles gradually increased in size, but there was almost no effect on soil particles with a particle size of 0.074 mm or less. As the slaking continued, the changes of particles with different particle sizes were mainly concentrated below 0.25 mm. Among the particle gradations corresponding to the highest point of strength of various contents, the most obvious change was the increase of the 0.25 mm to 0.1 mm particles from 15.25% to 22.59% in the soil samples with 7.5% CaO content, while 0.1 mm to 0.074 mm particles decreased from 8.72% to 2.17%. The specific gravity of the particles in the diameter range changed by 30%, which is almost 16% higher than the other group solutions, the number of particle contacts per unit volume became more, and the proportion of effective crystals deposited between particles became larger [28]. According to the rock-forming principle, like chemical and biological changes occur between the particles with the continuous slaking, CaCO_3_ deposits are deposited in the pores until solidification so that the particles gradually become agglomerated; the particle size becomes larger and can fill the tiny pores, forming CaCO_3_ bridges, which in turn play a reinforcing role and increase their strength. However, the increase in the particle gradation curve of the late slaking did not change significantly, proving that the effect may not be too satisfactory in over-slaking.

#### 3.3.2. Effect of Slaking Time on the Gradation of MgO Repair Materials Particles

**Figure 9:**
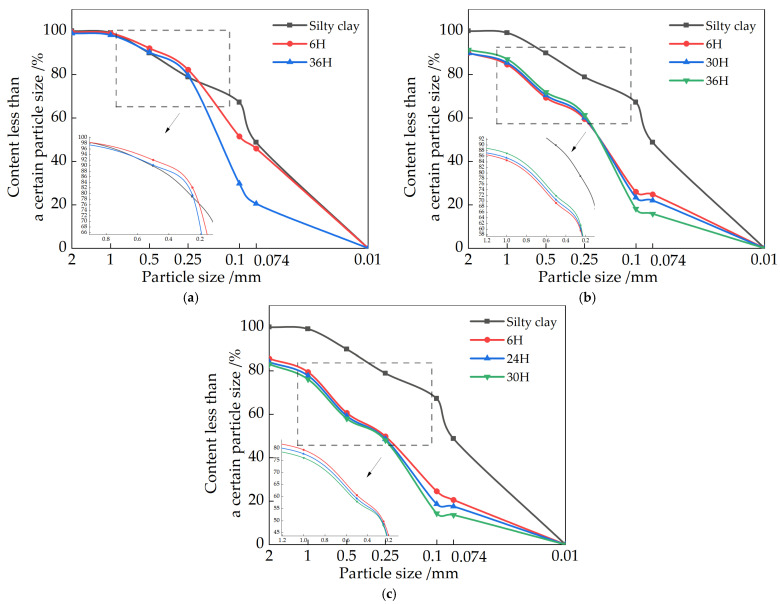
Particle gradation of repair materials with different MgO contents: (**a**) 7.5% MgO; (**b**) 15% MgO; (**c**) 22.5% MgO.

Figure 9 shows the particle gradation curves for different MgO contents with different slaking times. As shown in the figure, compared with the particle gradation curve of silty clay, the specific gravity of particles in the 0.25 to 0.1 mm particle size range increased from 11.64% to 32%, and the specific gravity of particles in the 0.1 to 0.074 mm particle size range decreased from 18% to 5.57% after 6h of slaking for the 7.5% MgO doping. However, the samples mixed with a higher content of MgO had particles larger than 2 mm in size in the early stage of slaking, and with the increase of the content, the large particles gradually increased. A rate of 22.5% MgO doping is more obvious, and the specific gravity of 0.5 mm to 2 mm particle size increased from 10.25% to 20.12%, whereas the specific gravity between 1 mm and 2 mm increased from 0.24% to about 5%, while the specific gravity below 0.074 mm decreased from 48.23% to 27.15%. It indicates that in the pre-slaking period, the high content of MgO has a relatively strong effect on the coarser and finer particles of the soil samples, and the fine particles gradually condense to form coarse particles. However, for the soil sample with 15% and 22.5% MgO, the sudden change in specific gravity of 0.25 mm to 0.074 mm particles occurred at the 30 h and 24 h, where the specific gravity of 0.25 mm to 0.1 mm particles increased from 26.49% to 43.18%. The 0.1 mm to 0.074 mm particles decreased from 5.12% to 0.34%, and those below 0.074 mm decreased from 27.45% to 17.25%. During this slaking time, the soil particles below 0.1 mm particle size condensed to 0.25 mm to 0.1 mm one by one, and there was almost no effect on the soil particles above the 0.25 mm particle size. As the slaking continued, the changes of particles with different particle sizes were mainly concentrated below 0.25 mm, and the trend was the same as before.

### 3.4. Microstructural Analysis

**Figure 10:**
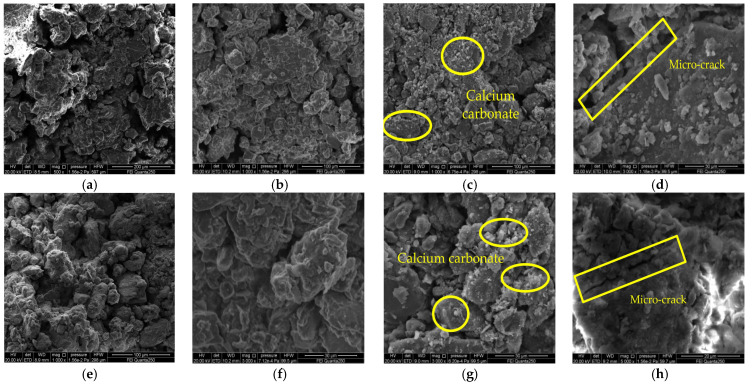
SEM images of CaO repair materials under the influence of slaking time: (**a**) silty clay (500×); (**b**) 15% CaO t = 6 h (1000×); (**c**) 15% CaO t = 48 h (1000×); (**d**) 15% CaO t = 54 h (3000×); (**e**) Silty clay (1000×); (**f**) 15% CaO t = 6 h (3000×); (**g**) 15% CaO t = 48 h (3000×); (**h**) 15% CaO t = 54 h (5000×).

The microstructure photos of the doped 15% CaO at different slaking times are shown in Figure 10. Through the microscopic pictures, it can be seen that the skeleton particles slaked for 6 h are mainly single grains with clear boundaries between the particles, a small amount of cementing material attached to the soil skeleton particles, and very few were located in the pores. In addition, there were some very unstable scaffold pores. Obvious nodular structures were also observed in the soil samples slaked for 6 h (Figure 10b,f), which indicated that a reaction of volcanic ash between the silty clay and lime was taking place, filling the pores between the particles and making soil samples denser, which explains the increase in strength of the lime at the early stage of slaking. After a period of slaking, the contours between the particles are difficult to see; the surface of the soil particles is attached by a large amount of cementing material, and the pores between the particles are filled. Figure 10c,g show the formation of irregular nanoscale amorphous calcium carbonate structures in the silty clay, similar to that observed by Cizer [29] et al. These calcium carbonates make the soil sample more monolithic and cause another increase in its strength. Figure 10d,h shows the formation of added calcium carbonate with a molar volume greater than that of the lime, swelling the soil–lime composite and the creation of microcracks [30]. It proves that over-slaking adversely affects the strength of the soil sample, which is the main reason for the reduction of strength in the later stages of slaking.

**Figure 11:**
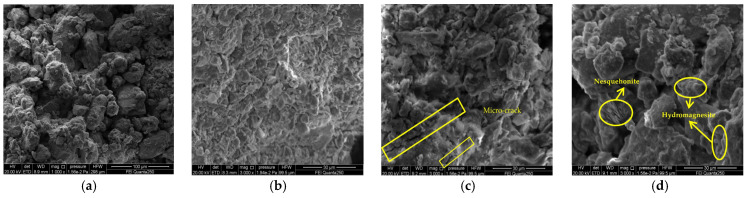
SEM images of MgO repair materials under the influence of slaking time: (**a**) silty clay (1000×); (**b**) 15% MgO t = 36 h (3000×); (**c**) 15% MgO t = 54 h (3000×); (**d**) 15% MgO t = 48 h (3000×).

Figure 11 shows the SEM (Scanning Electron Microscopy) images of soil samples before and after adding the MgO for slaking. After the slaking and carbonization reactions, nesquehonite and hydromagnesite appeared in the soil samples. In most of the samples, the microstructure is comprised of dense flakes with occasional rose-shaped hydromagnesite forming at the top. These magnesium carbonate particles glue the loose soil particles together, and the carbonation products fill the soil sample pores, gluing the soil particles together by face-to-edge and other contacts, densifying the microstructure of the soil sample [31]. Some products also extend into the interstices between soil particles, forming an overall skeletal structure with increased strength (Figure 11b). However, there are still large nesquehonite particles surrounded by hydrazine brucite in the soil [32]; thus, the hydration products are not completely carbonized, and microcracks will occur in the soil samples in the later stage of slaking (Figure 11c), which is the main reason for the decrease of strength in the later stage of slaking.

### 3.5. Relationship between Strength of Repair Material and pH Value

#### 3.5.1. Relationship between Strength of CaO Repair Material and pH Value

**Figure 12:**
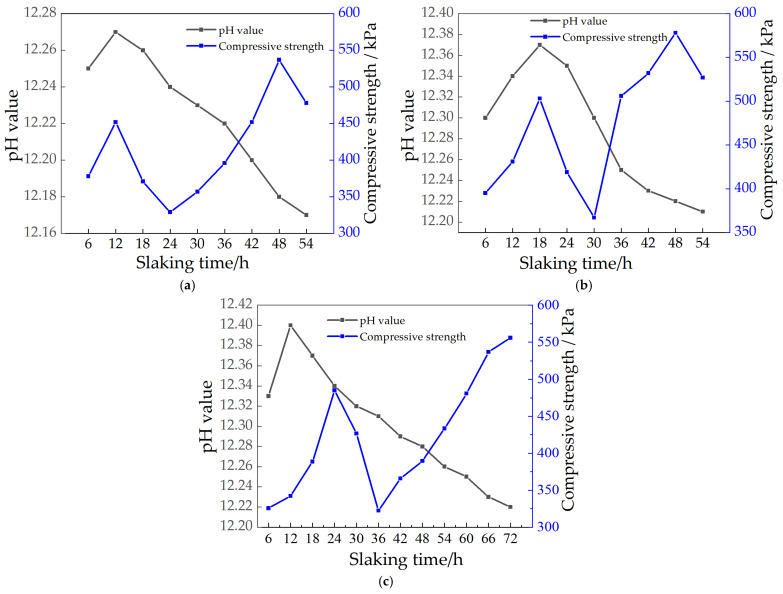
Relationship between pH value and strength of repair materials with different CaO contents: (**a**) 7.5%CaO; (**b**) 15%CaO; (**c**) 22.5%CaO.

Figure 12 shows the relationship curves between pH and compressive strength at different aging times for different CaO doping levels. From Figure 12, it can be found that there is some connection between the pH value and the compressive strength at different ageing times; both with increasing or decreasing pH value, the strength of the soil sample continuously changes. The relationship between the pH value and strength of all soil samples can be roughly divided into three segments: (1) Rising pH stage: corresponding to 12.25–12.27 for 7.5% CaO content, 12.30–12.37 for 15% CaO content, and 12.33–12.41 for 22.5% MgO content, with increasing compressive strength in this stage. (2) Repair material strength decline stage: 12.27~12.24 for 7.5% CaO content, 12.37~12.30 for 15% CaO content, and 12.33~12.31 for 22.5% CaO content, the pH value and compressive strength all decreased in this stage. (3) The pH decreased in the subsequent stages, and the strength then increased: considering the different amounts of CaO admixture, resulting in different types and contents of products under the same ageing time, the pH value and strength of different products varied. Therefore, the compressive strength of soil samples with different contents of CaO showed a trend of increasing then decreasing, and then increase with time, and the peak strength point has a greater lag than the peak pH point [33].

As the ageing continues to consume lime, a gel with low calcium to silicon ratio is formed, which facilitates the carbonation of the lime [34]. The carbonation products generated by the CaO-carbonated soil are expansive and continue to fill the pores between the soil particles, so when the soil sample is slaked for a period of time, the strength of the soil sample increases. However, with the passage of time, the water content of the mixed soil decreases, and although the expansive carbides will fill the pores and increase the strength, due to the low degree of reaction, the resulting calcium carbonate content is small, which is far from being able to compensate for the decrease in water content effect on strength. As the reaction continues, the amount of calcium carbonate produced increases, and its strength rises again due to the volume expansion of about 17% during the transformation of calcium hydroxide to calcium carbonate [35], making the soil more compact. However, the higher the lime content, the larger the gel pores of the reaction product [4], and more calcium carbonate can be filled, which is the main reason for the increase in strength of 22.5% doped CaO at 72 h of ageing. But as the reaction continues, the continued carbonisation of the calcium hydroxide wrapped around the carbonised product layer will lead to the expansion and cracking of the product layer, producing original microcracks leading to a reduction in strength.

#### 3.5.2. Relationship between Strength and pH Value of MgO Repair Materials

**Figure 13:**
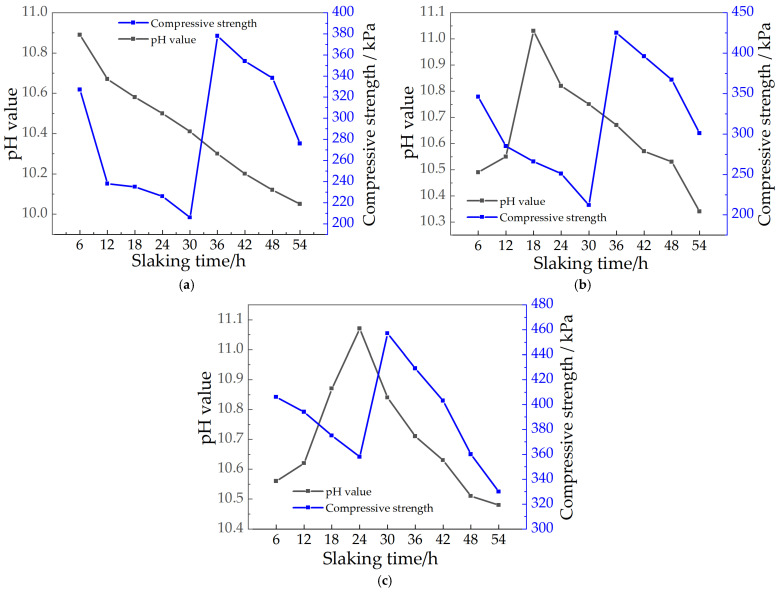
Relationship between pH value and strength of repair materials with different MgO contents: (**a**) 7.5% MgO; (**b**) 15% MgO; (**c**) 22.5% MgO.

Figure 13 shows the relationship curves between pH and compressive strength at different slaking times with different MgO doping. From Figure 13, we found that there is some connection between the pH value and compressive strength of the soil sample with different slaking times, and the strength of the soil sample changes with the change of pH value. The relationship between pH value and strength of all specimens can be roughly divided into three segments: (1) pH rising stage: corresponding to pH 10.51~11.12 for 15% MgO doping and 10.55~11.05 for 22.5% MgO doping, respectively, the strength of this stage decreases with the increase of pH. (2) Repair material strength rise stage: 10.41~10.31 for 7.5% MgO doping, 10.75~10.65 for 15% MgO doping and 11.05~10.84 for 22.5% doping, the strength will increase suddenly and reach the peak at this stage. (3) The pH value decreases, and the strength decreases in the subsequent stage. Considering the different dosages of MgO, the product content is different under the same slaking time, and the pH value and strength of different product content are different. However, the overall can be shown that the strength of soil samples with different contents of MgO showed a trend of decreasing at first and then increasing, and the peak point of strength also had a hysteresis compared with the peak point of pH value.

The main reasons for the above phenomenon are: MgO is an alkaline oxide, which reacts with water and hydrolyzes to produce OH^−^, making the pore solution alkaline, and when reacting with CO_2_ gas in the air, because CO_2_ is an acidic gas, it will ionize H^+^ and HCO^3−^ with water, making the pore solution pH lower. In this process, the soil sample is dominated by CO_2_ transport to promote the pore connectivity of carbonized soil. In the carbonization process, Mg^2+^ will react with CO_3_^2−^ and HCO_3_^−^ to generate magnesium carbonate, the carbonization product generated by MgO carbonized soil is an expansive product, which constantly fills the pores between these soil particles, so when slaked for a period of time, the strength of the soil sample will be improved, but when slaked for a period of time, the generation of more and more alkaline carbonate, in filling the gap at the same time, the stress generated by the volume expansion to produce cracks inside the soil sample continuously expand and develop; thus the strength of the soil sample is reduced.

## 4. Discussion

The mechanical properties of soil samples with different contents of CaO and MgO at various slaking times varied greatly. The underlying reason lies in the hydration and carbonization of MgO and CaO are continuous chemical reactions wherein different substances are generated at each stage. The pH value of the soil samples is increased when the added CaO and MgO combine with water forming Mg (OH)_2_ and Ca (OH)_2_. The expansion of molar volume during hydration is about 90% for CaO and 117% for MgO [36], however, the volume expansion of Ca (OH)_2_ to CaCO_3_ is about 17%. The volume expansion of the product at each stage contributes significantly to the mechanical responses of soil samples. In addition, nesquehonite and hydromagnesite, and basic calcium carbonate produced by the carbonization of MgO and CaO can fill the pores of the soil sample and bond the soil particles, which play a key role in improving the strength of soil samples. 

The strengths of the soil samples are basically consistent with the pH value and the particle size distribution. As mentioned in Section 5, there is a relationship between pH value and strength. For restoration materials with 15% and 22.5% CaO, it can be seen in Figure 9 that the strength of soil samples reaches its highest level in the pH range of 12.22~12.25 (98.5%~98.7% of the peak point) in the descending stage after the pH is reached. Similarly, as shown in Figure 10, the soil samples with 15% and 22.5% CaO reaches peak strength when the pH value is 10.55~10.70 (95.4%~97.7% of the peak point). On the other hand, the pH value also demonstrates the relationship between the slaking time and the particle size distribution of soil samples with restoration materials.

With the continuous progress of the hydration and carbonization reactions, the products, cement and the bonding degree between particles at each stage are various, resulting in changes in particle gradation and pH values. For soil samples with better particle gradation, the pore size is smaller, and the generated carbonate is more likely to cement adjacent particles together, resulting in more carbonate crystals adsorbed on the surface of the particles, increasing the deposition ratio of effective carbonate crystals, improving the strength of the soil samples. Therefore, the hydration reaction and product type of restoration materials can be reflected comprehensively and quickly by the pH value. However, in the actual situation, it is difficult to judge whether the pH is in the descending stage, so the percentage of the pH peak point can also be adopted to predict the strength of soil samples with restoration materials.

For soil samples with CaO, with the continuous carbonization of Ca(OH)_2_, the recrystallization process of calcite crystals from oblique morphology to rhombohedral morphology is the main reason for the increase in initial strength [37]. As the specific surface area of calcium hydroxide increases during the slaking process, the reactivity of Ca(OH)_2_ is greatly enhanced, and it can react with active SiO_2_ and Al_2_O_3_ in the soil samples in a short period of time. This reaction plays a role in cementing the soil particles, thereby increasing the strength of soil samples again and making the strength of soil samples appear to have two peak points.

Since tests in this work are carried out in a natural environment with low CO_2_ concentration, carbonization products and the denseness of soil samples after slaking are different from that of the accelerated carbonization test. More compact soil samples and more carbonization products in the accelerated carbonization test enhance the strength of soil samples [38], which explains the phenomenon of “lower soil strength compared with the accelerated carbonization test” observed in this experiment.

As the slaking time progresses, the increase of large soil particles will cause micro-cracks in soil samples, which will destroy the stability and integrity of the original soil structure. On the other hand, too-long slaking times lead to the evaporation of water and the failure to achieve the optimal moisture content of the soil, resulting in the low overall strength of soil samples.

There are many factors affecting slaking. Since the test is carried out indoors, it is affected by external factors such as indoor temperature and humidity, as well as human factors such as uneven mixing. The external environment that leads to the slaking of samples with different dosages changes or the additive content of a certain part of the sample is low, and the slaking is uneven, resulting in differences in the mechanical properties of different parts of the soil sample, and ultimately lead to deviations in the experimental results.

## 5. Conclusions

In this work, the properties of soil samples with different levels of lime and MgO are systematically investigated at the various slaking time; results are compared with soil samples without restoration materials under the same initial moisture content and maintenance conditions. From this study, the following conclusions were obtained:

(1) Slaking time exerts different effects on the pH value of soil samples with restoration materials. With the increase in slaking time, the pH value in the alkaline range of soil samples with higher content of lime and MgO shows a trend of first increasing and then decreasing. The soil sample containing 22.5% MgO and lime reach a pH peak of 11.12 within 24 h and a pH peak of 12.40 within 12 h, respectively, indicating that the carbonization reaction rate of MgO is slower than that of lime under natural conditions.
(2) With the increase of slaking time, the strength of soil samples containing MgO exhibits a trend of first decreasing and then increasing and finally decreasing. However, the strength of soil samples containing lime exhibits an opposite trend. Under a given moisture content (14.6%), the soil samples with MgO have a critical MgO content (20–25%) and a critical slaking time (24–36 h), which maximizes the strength of soil samples with restoration materials. Similarly, there exists a critical lime content (12.5–17.5%) and a critical slaking time (42–54 h) to maximize the strength of the soil samples containing lime at the moisture content of 14.6%.
(3) The microcracks generated by expansive carbonization products, including hydromagnesite crystals, magnesite carbon and calcium carbonate crystals, play an important role in the strength reduction of soil samples lime and MgO.
(4) The two oxides have different mechanisms for improving the properties of soil samples. The pH value of the soil samples with MgO is always lower than that of the soil samples with lime, and the optimal slaking time of the soil samples containing MgO is shorter. At low dosage, the soil samples containing lime display higher strength than soil samples containing MgO. At a dosage of 22.5%, the strength of soil samples containing MgO exhibits high strength within 48 h, and then the strength of soil samples containing lime gradually increases. Therefore, two kinds of restoration materials should be reasonably selected according to actual engineering; the differences in their respective reaction processes can be fully utilized to improve the mechanical properties of site soil.

The effects of restoration materials are not only affected by the dosage and slaking time but also by the initial moisture content and the slaking temperature and humidity. This paper only discusses the effect of the slaking time on the pH value and the strength of soil samples with restoration materials. Next, the influences of initial moisture content and slaking conditions on soil samples with restoration materials will be studied in further work.

## Figures and Tables

**Table 1:** Physical indexes of test soil samples.

Soil Type	Liquid Limit/%	Plastic Limit/%	Plasticity Index	Maximum Dry Density g/cm^3^	Optimum Moisture Content/%	Natural Moisture Content/%	pH	Compressive Strength/kPa
silty clay	37.63	21.03	16.6	1.68	14.76	12.5	9.26	372

**Table 2:** Experimental design.

Number	Variable		
	Additive	Content	Slaking Time
01	CaO	7.5%	6 h, 12 h, 18 h, 24 h, 30 h, 36 h, 42 h, 48 h, 54 h
02	MgO		
03	CaO	15%	6 h, 12 h, 18 h, 24 h, 30 h, 36 h, 42 h, 48 h, 54 h
04	MgO		
05	CaO	22.5%	6 h, 12 h, 18 h, 24 h, 30 h, 36 h, 42 h, 48 h, 54 h, 60 h, 66 h, 72 h
06	MgO		
07	-	-	6 h, 12 h, 18 h, 24 h, 30 h, 36 h, 42 h, 48 h, 54 h

## Data Availability

The data presented in this study are available on request from the corresponding author.
